# Risk of second primary breast cancer among cancer survivors: Implications for prevention and screening practice

**DOI:** 10.1371/journal.pone.0232800

**Published:** 2020-06-04

**Authors:** Yao Cheng, Ziming Huang, Qing Liao, Xingchen Yu, Hongyu Jiang, Yangting He, Shuang Yao, Shaofa Nie, Li Liu

**Affiliations:** 1 Maternal and Child Health Hospital of Hubei Province, Tongji Medical College, Huazhong University of Science and Technology, Wuhan, Hubei, China; 2 Department of Epidemiology and Biostatistics, Ministry of Education Key Lab of Environment and Health, School of Public Health, Tongji Medical College, Huazhong University of Science and Technology, Wuhan, Hubei, China; Kaiser Permanente Washington Health Research Institute, UNITED STATES

## Abstract

Second primary breast cancer (SPBC) is becoming one of the major obstacles to breast cancer (BC) control. This study was aimed to determine the trend of SPBC incidence over time and the risk of developing SPBC in site-specific primary cancer survivors in the United States. The Surveillance, Epidemiology, and End Results (SEER) 13 registry (1992–2015) was used to identify SPBC patients with previous malignancies. Standardized incidence ratio (SIR) was computed to compare the incidence rates of the observed cases of SPBC in cancer survivors over the expected cases in the general population. Elevated risk of SPBC was observed in women with previous BC (SIR = 1.74) or thyroid cancer (SIR = 1.17). Women with initial skin melanoma in older age (≥50 years) (SIR = 1.11), or White race (SIR = 1.11) presented an elevated incidence of SPBC than the general female population. Besides, Asian/Pacific Islander (API) women with cancer of corpus uteri, ovary, bladder, or kidney were prone to developing SPBC when compared with the general population, with SIRs of 1.61, 1.35, 1.48, and 1.70, respectively. Male BC patients showed profound risk of developing SPBC (SIR = 34.86). Male leukemia patients also presented elevated risk of developing SPBC (SIR = 2.06). Our study suggests significant increase of SPBC in both sexes in the United States. Elevated risk of SPBC exists in survivors with primary BC, female thyroid cancer, male leukemia, and API female cancer patients with primary genitourinary cancer. Our study is helpful in developing strategies for BC control and prevention on specific first primary cancer survivors with an elevated risk of SPBC.

## Introduction

Breast cancer (BC) is one of the most common malignancies worldwide. Globally, multiple strategies have been applied to reduce the disease burden of BC. Along with the promotion of BC screening and treatment, the incidence and mortality of BC in the United States have been decreasing in recent years [1]. However, the prognosis of cancer patients combined with BC was still concerning. Besides, the improvement of early detection and treatment technology could extend the life of cancer patients to a great extent in the future. The number of cancer survivors is estimated to reach 20.3 million by 2026 in the United States [2]. As survival improves, the accumulated first primary cancer patients may heighten the time interval needed for the development of second primary breast cancer (SPBC), which is becoming a growing obstacle to BC control [3, 4].

Considering the elevated risk of SPBC among female survivors with primary BC [5, 6], the American Cancer Society (ACS) and the National Comprehensive Cancer Network (NCCN) recommend that women undergoing a unilateral mastectomy or lumpectomy should be followed with annual mammography [7]. Besides BC, previous studies have observed elevated risks of SPBC among female patients with initial skin melanoma [8], thyroid cancer [9, 10], and ovarian cancer [11]. However, national guidelines on SPBC prevention or screening for cancer survivors remain limited. And the links between other primary cancers and the risk of subsequent SPBC is limited and controversial [12, 13]. Furthermore, this disparity of ethnicities and sex on subsequent risk of SPBC is still unknown.

Since the risk of developing SPBC in primary cancer survivors has not been well studied, we sought to investigate the trends of SPBC incidence over time and to explore the risk of developing SPBC among various site-specific cancer survivors stratified by sex, race, age, latency period, and treatment in the United States using the Surveillance, Epidemiology, and End Results (SEER) database.

## Materials and methods

### Study site and population

The National Cancer Institute’s SEER 13 database, a wildly used open-access cancer registry, covering approximately 13.4% of the US population from San Francisco-Oakland, Connecticut, Metropolitan Detroit, Hawaii, Iowa, New Mexico, Seattle/Puget Sound, Utah, Metropolitan Atlanta, San Jose-Monterey, Los Angeles, and rural Georgia was used in this study [14]. Site-specific invasive cancer cases were identified according to the International Classification of Disease for Oncology, 3^rd^ Revision. Among the first primary cancer cases (3,798,691), those with a death certificate only (44,518), zero follow-up time (17,972) or missing follow-up time (15,503), and those survived less than 6 months (623,525) were excluded. Then, ductal or invasive SPBC diagnosed within 6 months of first primary cancer diagnosis (601) were further excluded as these were likely to be preexisting or synchronous cancers. The end of the follow-up was the date of the second primary cancer diagnosis, death, or most updated date of the dataset (December 31, 2015), whichever occurred first. Finally, a total of 1,566,538 female and 1,530,034 male initial primary cancer cases at risk for an SPBC were registered in SEER 13 between 1992 and 2015. The 20 most common first primary cancer sites in the United States in 2018 were selected, including oral cavity and pharynx, stomach, colon and rectum, liver and intrahepatic duct, pancreas, lung and bronchus, melanoma of the skin, breast, cervix uterus, corpus uteri, ovary, prostate, testis, bladder, kidney, central nervous system, thyroid, non-Hodgkin lymphoma (NHL), myeloma, and leukemia [15].

### Statistical analysis

We compared the demographic characteristics of first primary breast cancer (FPBC) patients and SPBC patients, including age, race, stage, grade, size, the status of estrogen receptor **(**ER) and progesterone receptor **(**PR), radiotherapy, and survival rates. Stage of BC was defined according to the American Joint Committee on Cancer 6^th^ staging system. Survival rates were classified by the proportion of cancer patients survived ≤1 year, 1–5 years, 5–10 years, and >10 years. The difference between patient characteristics of FPBC and SPBC was computed by Chi-square tests.

The age-standardized incidence rates of SPBC and FPBC were calculated by direct standardization, using the standard population revealed by the World Health Organization (WHO 2000–2025). Trends in incidence of SPBC were calculated using the Joinpoint regression analysis which was proposed by Kim et al (Joinpoint Regression Software, Version 4.0.4-May 2013; Statistical Methodology and Applications Branch, Surveillance Research Program of the US National Cancer Institute) [16]. The average annual percentage changes (AAPCs) with the corresponding 95% confidence intervals (CIs) were further estimated.

Standardized incidence ratio (SIR) and the corresponding 95% CI were used to compare the incidence rates of the observed cases of SPBC in cancer survivors over the expected cases in the general population [17]. The observed cases of SPBC were those developed BC after at least 6 months of the diagnosis of first primary cancer. The expected cases of SPBC were numbers in FPBC survivors calculated by BC risk in the general population with no cancer history. The subgroups analyses of SIR were further stratified by sites of first primary cancer, sex (female, male), age at diagnosis of first primary cancer (<50, ≥50 years), race [White, Black, and Asian/Pacific Islander (API)], latency period after first primary cancer diagnosis (6–11, 12–59, 60–119, ≥120 months), and treatment (with radiotherapy, without radiotherapy). The SIRs and corresponding 95% CIs were calculated by the software of SEER*Stat MP-SIR, developed by the National Center for Health Statistics and the Census Bureau (https://seer.cancer.gov/seerstat/). Two-sided *P* values less than 0.05 were considered as statistically significant.

## Results

There were 49,587 female and 546 male SPBC cases observed during 1992–2015, accounting for 3.17% and 0.04% of the first primary cancer survivors, respectively. The characteristics of FPBC and SPBC are shown in Table 1. Compared with FPBC patients, SPBC patients were more likely to be White race, older (≥50 years), localized, better differentiated, hormone receptor-positive, and to have a smaller tumor size. However, the 10-year survival rate was significantly lower in SPBC patients than FPBC patients (17.18% *vs* 36.63%, *P*<0.0001).

**Table 1:** Demographic characteristics of first primary and second primary breast cancer among female and male patients.

	Female				Male			
	FPBC (%)	SPBC (%)	χ^2^	*P*	FPBC (%)	SPBC (%)	χ^2^	*P*
Age								
<50	137175 (24.93)	5430 (10.95)	4903.31	<0.0001	435 (11.84)	11 (2.01)	48.52	<0.0001
≥50	413146 (75.07)	44157 (89.05)			3240 (88.16)	535 (97.99)		
Race								
White	436933 (79.4)	40272 (81.21)	406.15	<0.0001	2921 (79.48)	445 (81.50)	-	-
Black	53132 (9.65)	4913 (9.91)			474 (12.90)	70 (12.82)		
American Indian/Alaska Native	3858 (0.70)	193 (0.39)			9 (0.24)	0 (0)		
Asian/Pacific Islander	53322 (9.69)	4194 (8.46)			238 (6.48)	30 (5.49)		
Unknown	3076 (0.56)	15 (0.03)			33 (0.90)	1 (0.18)		
Stage								
Localized	254266 (46.20)	32674 (65.89)	10223.94	<0.0001	1299 (35.35)	263 (48.17)	75.87	<0.0001
Regional	128286 (23.31)	10836 (21.85)			1241 (33.77)	200 (36.63)		
Distant	20790 (3.78)	2194 (4.42)			224 (6.10)	35 (6.41)		
Unknown	146979 (26.71)	3883 (7.83)			911 (24.79)	48 (8.79)		
Grade								
Well differentiated	98380 (17.88)	10442 (21.06)	1148.21	<0.0001	419 (11.40)	63 (11.54)	4.95	0.2925
Moderately differentiated	200692 (36.47)	19928 (40.19)			1598 (43.48)	254 (46.52)		
Poorly differentiated	167285 (30.40)	13923 (28.08)			1101 (29.96)	163 (29.85)		
Undifferentiated	8148 (1.48)	493 (0.99)			56 (1.52)	4 (0.73)		
Unknown	75816 (13.78)	4801 (9.68)			501 (13.63)	62 (11.36)		
Status								
ER+/PR+	308212 (56.01)	28929 (58.34)	1063.42	<0.0001	2486 (67.65)	406 (74.36)	13.57	0.0088
ER+/PR-	55831 (10.15)	6367 (12.84)			305 (8.30)	45 (8.24)		
ER-/PR+	8434 (1.53)	594 (1.20)			32 (0.87)	2 (0.37)		
ER-/PR-	87278 (15.86)	7941 (16.01)			137 (3.73)	19 (3.48)		
Unknown	90566 (16.46)	5756 (11.61)			715 (19.46)	74 (13.55)		
Size								
≤1 cm	346099 (62.89)	41184 (83.05)	877.54	<0.0001	2189 (59.56)	435 (79.67)	13.98	0.0002
1-4cm	149732 (27.21)	6351 (12.81)			1154 (31.40)	98 (17.95)		
>4cm	22948 (4.17)	766 (1.54)			137 (3.73)	4 (0.73)		
Unknown	31542 (5.73)	1286 (2.59)			195 (5.31)	9 (1.65)		
Radiotherapy								
Yes	263011 (47.79)	17082 (34.45)	3260.26	<0.0001	915 (24.90)	111 (20.33)	-	-
No	5553 (1.01)	569 (1.15)			39 (1.06)	0 (0)		
Unknown	281757 (51.20)	31936 (64.4)			2721 (74.04)	435 (79.67)		
Survival								
≤1 year	55716 (10.12)	7496 (15.12)	8665.49	<0.0001	511 (13.90)	96 (17.58)	56.69	<0.0001
1–5 years	155088 (28.18)	20235 (40.81)			1277 (34.75)	259 (47.44)		
5–10 years	134515 (24.44)	13212 (26.64)			957 (26.04)	111 (20.33)		
>10 years	201594 (36.63)	8521 (17.18)			907 (24.68)	75 (13.74)		
Unknown	3408(0.62)	123(0.25)			23 (0.63)	5 (0.92)		

Abbreviations: FPBC: first primary breast cancer; SPBC: second primary breast cancer; ER: estrogen receptor; PR: progesterone receptor.

**Fig 1:**
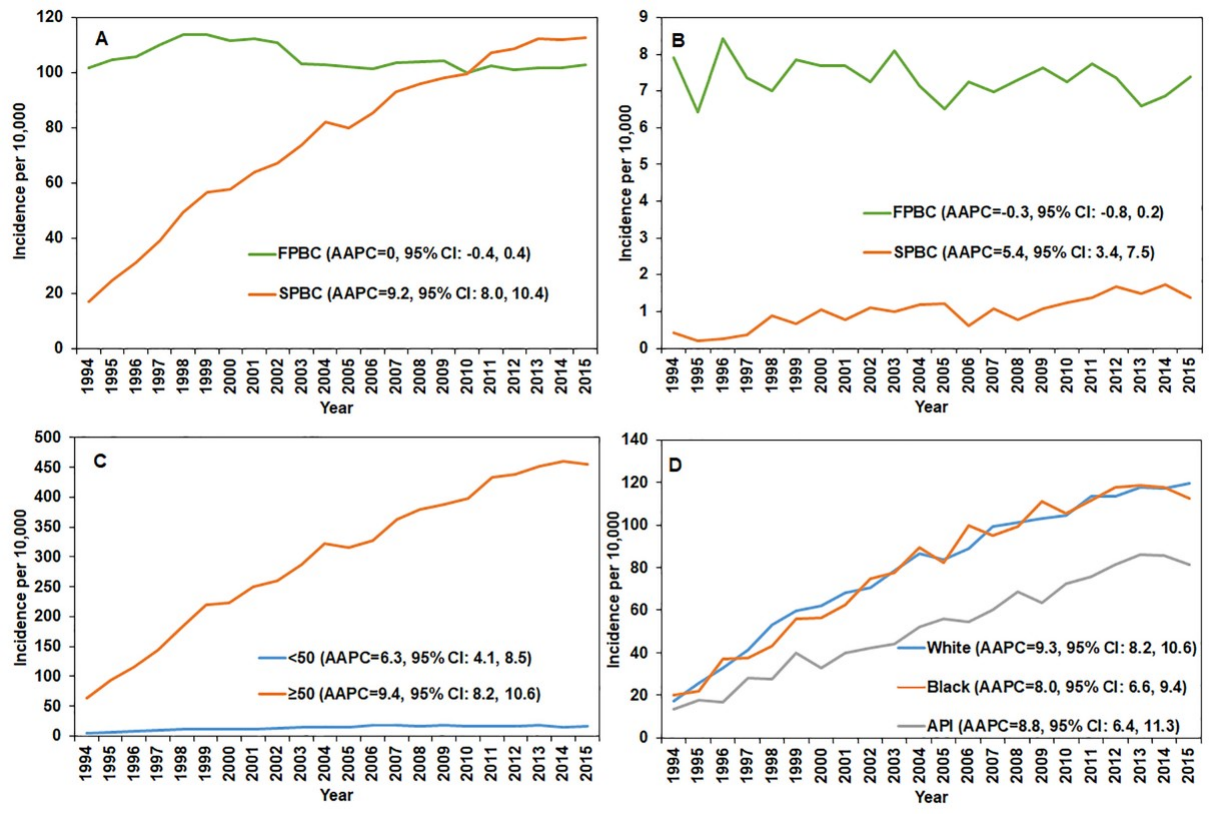
Trends in age-standardized rates of first primary and second primary breast cancer patients. Abbreviations: AAPC: average annual percentage change; API: Asian/Pacific Islander; FPBC: first primary breast cancer; SPBC: second primary breast cancer. Age-standardized rates of first primary per 100,000 and second primary breast cancer per 1,000,000 for female (A) and male (B), and age-standardized rates of second primary breast cancer per 1,000,000 for women stratified by age (C) and race (D).

Considering the small number of SPBC cases attributable to the short-latency in the first two years, the trend analyses of SPBC in both sexes started in 1994. The trends of FPBS and SPBC between 1994 and 2015 are presented as Fig 1A and 1B. The incidence of SPBC significantly increased during 1994–2015 in female and male cancer survivors, with AAPCs of 9.2% (95% CI: 8.0%-10.4%) and 5.4% (95% CI: 3.4%-7.5%), respectively. When stratified by age and ethnicity, all of the subgroups showed upward trends, with AAPCs ranging from 6.3% to 9.4% (Fig 1C and 1D).

Furthermore, the trends in age-standardized incidence of first primary cancers of both sexes stratified by ethnicity were provided in S1–S4 Figs. And the trend analyses of first primary cancers during 1992–2015 were showed in S1 Table. In the male population, the incidence of oral cavity and pharynx, stomach, colorectal, lung and bronchus, prostate, bladder, central nervous system, NHL, and leukemia presented significant downward trends, with AAPCs ranging from -0.7% to -3.8%. However, the incidence of liver and intrahepatic duct, pancreas, melanoma of the skin, testis, kidney, thyroid, and myeloma showed significant increasing trends, with AAPCs ranging from 0.3% to 3.0%. For Female patients, downward trends were observed in the incidence of oral cavity and pharynx, stomach, colorectal, lung and bronchus, cervix uterus, ovary, bladder, central nervous system, and leukemia, with AAPCs ranging from -0.3% to -1.8%. Additionally, significant upward trends were observed in the incidence of liver and intrahepatic duct, pancreas, melanoma of the skin, kidney, and thyroid cancer, with AAPCs ranging from 0.3% to 4.5%.

Overall, initial cancer survivors had significantly elevated risks of developing SPBC in both sexes (SIR = 1.35, 95% CI: 1.34–1.36; SIR = 1.16, 95% CI: 1.06–1.26, respectively) compared with the general population (Table 2). Significant differences of the subsequent SPBC were found between patients with or without breast initial cancer. BC in females (61.85%) and prostate cancer in males (43.33%) were the most common first primary cancers for subsequent SPBC, respectively. Further analyses by cancer sites revealed that patients with BC, thyroid cancer, melanoma of the skin, and corpus uteri cancer presented significantly elevated risks of SPBC when compared with the general population, with SIRs of 1.74 (95% CI: 1.72–1.76), 1.17 (95% CI: 1.10–1.23), 1.08 (95% CI: 1.04–1.12) and 1.05 (95% CI: 1.01–1.09), respectively. Male survivors with initial BC or leukemia were at higher risk of developing SPBC when compared with the general male population, with SIRs of 34.86 (95% CI: 25.23–46.95) and 2.06 (95% CI: 1.20–3.29), respectively.

**Table 2:** Risk of second primary breast cancer after previous malignancy stratified by initial primary site and sex.

Initial primary site			Female			Male
	Observed	Expected	SIR (95%CI)	Observed	Expected	SIR (95%CI)
Oral cavity and pharynx	412	451.72	0.91 (0.83, 1.00)	12	10.02	1.20 (0.62, 2.09)
Stomach	163	202.23	0.81 (0.69, 0.94)	5	3.49	1.43 (0.47, 3.35)
Colon and rectum	3370	3530.05	0.95 (0.92, 0.99)	54	47.72	1.13 (0.85, 1.48)
Liver and intrahepatic duct	46	62.64	0.73 (0.54, 0.98)	0	-	-
Pancreas	88	101.89	0.86 (0.69, 1.06)	3	1.14	2.63 (0.54, 7.70)
Lung and bronchus	1138	1241.28	0.92 (0.86, 0.97)	19	13.86	1.37 (0.83, 2.14)
Melanoma of the skin	2551	2366.22	1.08 (1.04, 1.12)	43	35.58	1.21 (0.87, 1.63)
Breast	30795	17674.09	1.74 (1.72, 1.76)	43	1.23	34.86 (25.23, 46.95)
Cervix uterus	487	631.46	0.77 (0.70, 0.84)	-	-	-
Corpus uteri	3026	2877.83	1.05 (1.01, 1.09)	-	-	-
Ovary	925	915.25	1.01 (0.95, 1.08)	-	-	-
Prostate	-	-	-	237	254.32	0.93 (0.82, 1.06)
Testis	-	-	-	2	1.72	1.17 (0.14, 4.21)
Bladder	741	775.35	0.96 (0.89, 1.03)	36	33.01	1.09 (0.76, 1.51)
Kidney	687	653.29	1.05 (0.97, 1.13)	14	12.07	1.16 (0.63, 1.95)
Central nervous system	98	112.32	0.87 (0.71, 1.06)	2	0.94	2.13 (0.26, 7.70)
Thyroid	1321	1132.30	1.17 (1.10, 1.23)	7	3.39	2.07 (0.83, 4.26)
Non-Hodgkin lymphoma	1123	1222.42	0.92 (0.87, 0.97)	20	14.43	1.39 (0.85, 2.14)
Myeloma	176	235.89	0.75 (0.64, 0.86)	2	3.37	0.59 (0.07, 2.14)
Leukemia	440	501.26	0.88 (0.80, 0.96)	17	8.26	2.06 (1.20, 3.29)
All sites	49788	36911.39	1.35 (1.34, 1.36)	547	472.88	1.16 (1.06, 1.26)
All sites except for breast	18993	19237.30	0.99 (0.97, 1.00)	504	471.65	1.07 (0.98, 1.17)

Abbreviations: SIR: standardized incidence ratio; CI: confidence interval.

**Fig 2:**
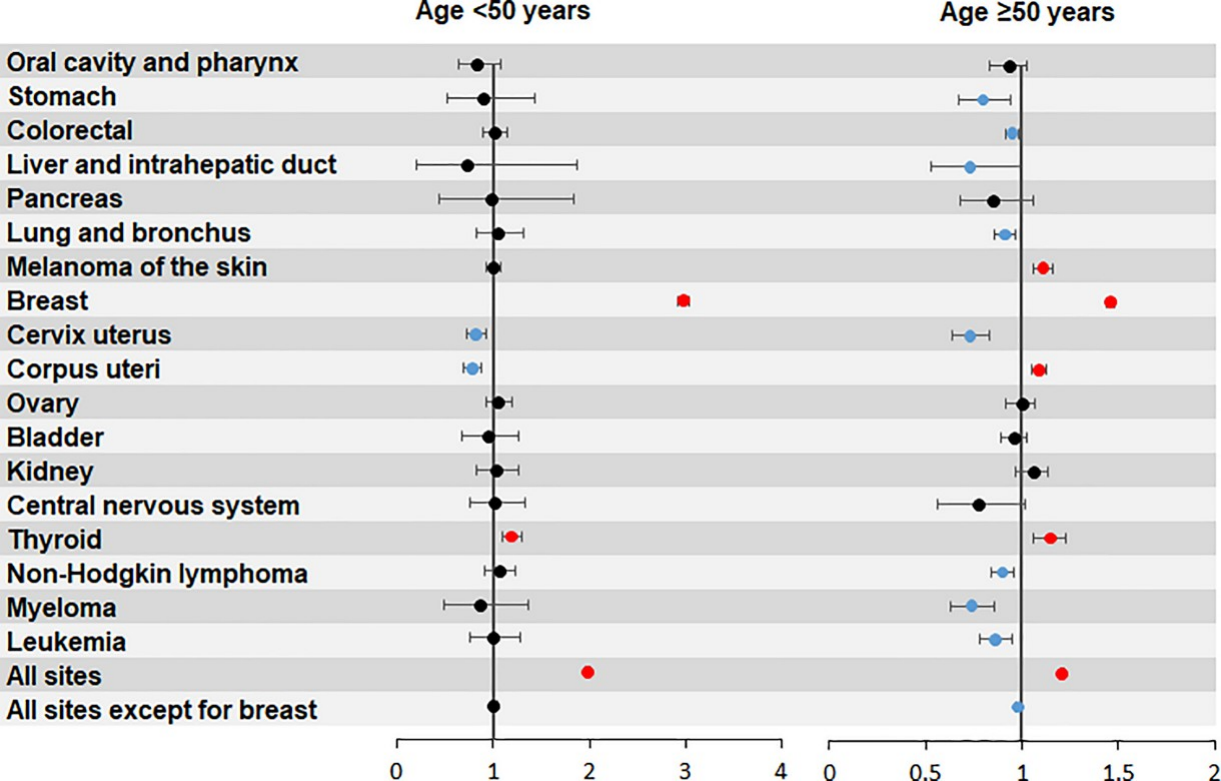
Risk of female second primary breast cancer after previous malignancy stratified by initial primary site and age group.

The SIRs for female SPBC stratified by age are presented in Fig 2. Compared with older cancer patients (≥50 years), the younger ones (<50 years) harbored a higher risk of developing SPBC. Female patients with BC or thyroid cancer harbored elevated risk of subsequent SPBC in both age groups, with SIRs of 2.98 (95% CI: 2.92–3.04) and 1.19 (95% CI: 1.10–1.30) in younger age group, and 1.46 (95% CI: 1.44–1.48) and 1.15 (95% CI: 1.06–1.23) in older age group, respectively. In addition, the risk of developing SPBC was significantly higher in older female patients with previous melanoma of the skin (SIR = 1.11, 95% CI: 1.06–1.16), or corpus uteri cancer (SIR = 1.09, 95% CI: 1.05–1.13) compared with the general population.

**Fig 3:**
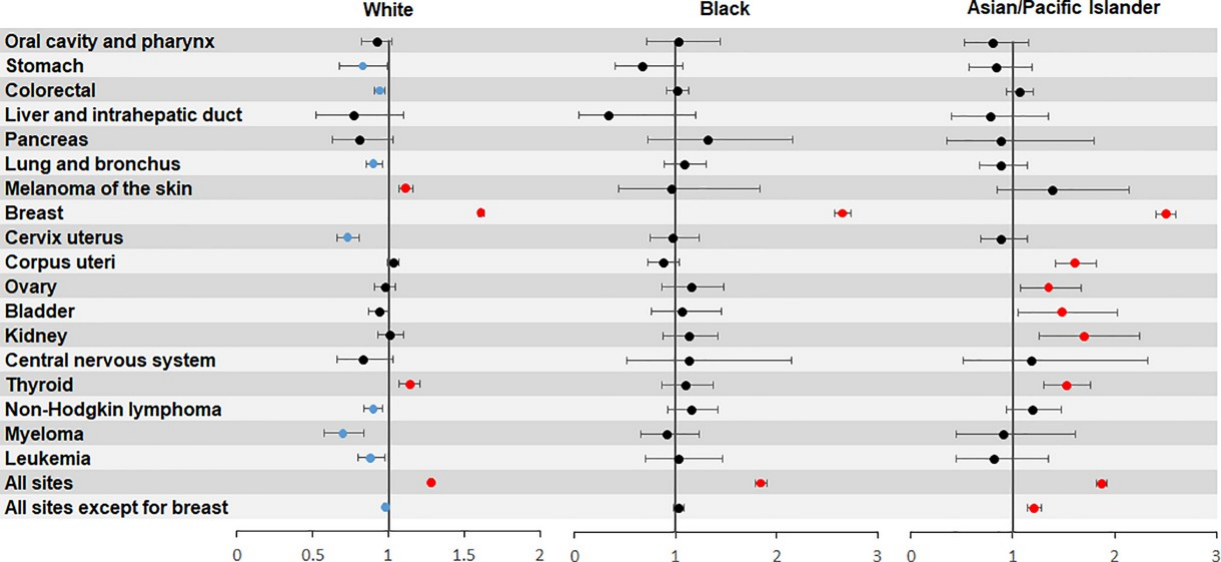
Risk of female second primary breast cancer after previous malignancy stratified by initial primary site and race.

Stratified analyses by race (White, Black, and API) showed elevated risks of SPBC among female cancer survivors across the three populations (Fig 3). The SIRs of developing SPBC in female FPBC patients ranged from 1.61 to 2.65 in the three ethnicities. In addition, the white women with primary skin melanoma or thyroid cancer were at elevated risk of developing SPBC (SIR = 1.11, 95% CI: 1.07–1.16; SIR = 1.14, 95% CI: 1.07–1.21, respectively). Elevated risk of subsequent SPBC was also observed in API female patients with non-breast primary cancer (SIR = 1.21, 95% CI: 1.15, 1.28), especially among patients with corpus uteri cancer (SIR = 1.61, 95% CI: 1.42–1.82), ovarian cancer (SIR = 1.35, 95% CI: 1.08–1.67), bladder cancer (SIR = 1.48, 95% CI: 1.05–2.03), kidney cancer (SIR = 1.70, 95% CI: 1.26–2.24), or thyroid cancer (1.53, 95% CI: 1.26–2.24).

The risk of SPBC among female cancer patients by follow-up period is shown in S2 Table. Elevated SIRs were observed in all four latency periods among FPBC patients, and the SIRs increased with latency periods. For female patients with skin melanoma, increased risk of SPBC was noticed within 1–10 years after primary diagnosis. Elevated SIRs were also found among female patients with corpus uteri cancer during the follow-up period between 6 months and 10 years. After a 1-year latency of thyroid diagnosis, the observed risk of SPBC was higher than expected. The risk of SPBC increased after 10 years of primary diagnosis of NHL.

Increased SIRs were found among female patients with FPBC or thyroid cancer regardless of radiation treatment (S3 Table). Besides, female patients with primary skin melanoma or corpus uteri cancer receiving no radiation therapy were at higher risk of developing SPBC when compared with the general population.

## Discussion

During 1994–2015, the incidence of female and male SPBC in the United States increased by 9.2% and 5.4% per year, respectively. Compared with the general population, the sex-specific risk of developing SPBC among female and male cancer survivors rose by 35% and 16%, respectively. In addition, female patients with FPBC, skin melanoma, thyroid cancer, corpus uteri cancer; and male patients with FPBC, leukemia were at elevated risk of developing SPBC. On the other hand, female patients with stomach, colon and rectum, liver and intrahepatic duct, lung and bronchus, cervix uterus, non-Hodgkin lymphoma, myeloma, and leukemia were at lower risk of SPBC. Furthermore, younger cancer survivors had a higher risk of subsequent SPBC compared with the elders. Besides, compared with the API general population, the non-breast primary female patients were also at an elevated risk of developing SPBC. To our knowledge, this is the first comprehensive study that identified various site-specific cancer survivors at elevated risk of SPBC, which could offer convincible evidence for further BC prevention and screening practice on targeted first primary cancer survivors with elevated risks of SPBC.

The higher than expected incidence of SPBC might be partly explained by the greater medical surveillance among cancer survivors than the general population. It is worth noting that the risk of developing SPBC is much elevated in male FPBC patients than female FPBC cases. FPBC has been confirmed as a risk indicator for SPBC in women [6, 18]. Besides, previous studies have reported a 30%-52% increased risk of developing SPBC in male primary BC survivors when compared with the general population [19–21]. Prolonged survival associated with early diagnosis and definitive surgery and therapy plays a role in the increased risk of SPBC in both sexes. Apart for the shared similar risk factors such as hormonal, familial, and genetic risk factors in female and male BC patients [22], additional risk factors such as Klinefelter syndrome, gynaecomastia, and testicular disease might have partly contributed to the profound elevated risk for SPBC in male BC patients [23, 24]. Also, this might be a function of the impact of hormonal therapy interacting with the androgen/estrogen profile in men versus women. Moreover, men are more prone to have drinking or smoking behavior than women, which might partly have contributed to the sharp increase trend of SPBC in men.

In accordance with the present results, previous studies have reported an elevated incidence of SPBC in thyroid cancer survivors [9, 10]. Potential mechanisms for the association between thyroid cancer and subsequent BC include the shared common risk factors of hormone, genetics, and environment. One of the molecular pathogenesis of the elevated risk of SPBC in thyroid cancer patients could be the high ER levels in thyroid cancer patients compared with the general population in whom sex steroid receptors are present in human thyroid tissue [25, 26]. It should be noted that the incidence of first primary thyroid cancer showed significantly increasing trends. Thus, the SPBC control among thyroid cancer survivors should be strengthened.

The significantly increased risk of SPBC (SIR = 1.08) among female skin melanoma patients is consistent with previous findings (SIRs from 1.07 to 4.10) [27–30]. Cutaneous melanoma and BC share common mutations at susceptibility genes, like *BRCA2* and *CDKN2A* [31]. Besides, both melanoma and BC cells exhibit ERs. It is possible that hormonal factors in melanoma cancer survivors promote the development of SPBC [32]. In addition, melanoma and BC frequently happened among women with high socioeconomic status, which might be another reason for the increased risk of SPBC in melanoma survivors [33]. Race stratified analysis revealed that only white patients with melanoma were at increased risk of SPBC, which could be due to the high incidence of skin melanoma in white women but less often in the other two races. Moreover, the increasing incidence of skin melanoma also might be partly attributed to the upward trend of the following SPBC. Besides, the elevated risk of SPBC among female melanoma was only observed in older patients but not in younger ones. Although the mechanism is not well known, it is referred that later menopausal (≥50 years) melanoma patients might have an increased risk of developing BC, and postmenopausal estrogen therapy for melanoma survivors may also lead to a higher risk of SPBC than the general population.

This study found close associations between primary gynecological cancers and SPBC, especially in the API population. API women with a previous history of ovarian or corpus uteri cancer presented an elevated risk of SPBC, which is in agreement with previous studies conducted in the Asian population from Taiwan [11, 13]. It might be partly explained by the higher survival rate among API ovarian or corpus uteri cancer patients. Several of the underlying mechanisms of the ovary-breast link have been addressed previously. Patients carrying mutations in *BRCA* or *TP53* have significantly increased lifetime risk for developing breast and ovarian cancer [34, 35]. Nonetheless, due to the lack of optimal genetic testing in Asian ovarian cancer patients [36], much less is known regarding the mechanisms of the race effect on the ovary-breast link. Further research is needed to clarify the biological and environmental mechanisms on the monotonically elevated risk of SPBC following corpus uteri or ovary cancer among the API population.

Furthermore, the elevated risk of SPBC in previous kidney or bladder cancer patients was also observed in API women. The kidney-breast cancer link was inconsistent in previous publications. Murray et al recently found a lower than expected rate of BC in renal cortical neoplasms using a 90 day of latency [37]. While a study based on the European Prospective Investigation into Cancer and Nutrition cohort indicated the kidney-breast association by revealing an excessive risk of kidney cancer among BC survivors [38]. However, no study has reported the increased risk of SPBC in patients with kidney cancer until now. For bladder cancer, this study is in line with a previous study that observed an elevated risk of BC in female patients with first primary bladder cancer in Korea [39]. It was hypothesized that fumarate hydratase germline mutations in bladder and BC patients might partly contribute to the phenomenon [40]. Additionally, the upward trends in the incidence of first primary bladder and kidney cancer reinforced the benefit to lower first primary cancers in BC control. Further studies are needed to clarify the mechanisms of increased risk of SPBC among API female patients with kidney or bladder cancer.

The risk of SPBC significantly elevated after 10 years of primary NHL diagnosis, which is in agreement with a study conducted by Bluhm et al [41]. It might be attributable to the long-term effects of radiotherapy, or the combination of radiotherapy and chemotherapy of NHL.

As for male cancer survivors, leukemia survivors had an elevated risk of SPBC. This finding is novel, as BC has not been reported to occur more frequently after the first primary leukemia diagnosis in the previous studies. This could potentially highlight the similarity in disease biology in male leukemia and BC.

Our study is based on the SEER database, which is one of the largest cancer registries and enables adequate analysis of multiple malignancies during the long-term follow-up period. Our study is of importance since we supplied the specific initial cancers with elevated risks of SPBC by using the most recent data with a large number of cases. This study is novel because many previous studies did not stratify according to race and we revealed that API cancer patients possessed a higher risk of developing SPBC when compared with other ethnicities in the United States. Moreover, this study combined the trends in incidence of site-specific first primary cancers with the expected risk of SPBC, which could offer more evidence for the identification of high-risk groups on SPBC prevention and control.

The present study has several limitations. First, first primary cancer patients of different ages with different types and severity of cancer could have varying life expectancy to develop SPBC, which might undermine the generalization of the results. However, with the development of medical treatment, the prolonging life-span of all kinds of cancer patients may increase the risk of developing SPBC. Thus, the result of this study still emphasized the SPBC control of specific cancer survivors. Second, the first primary cancer patients who have moved out of the 13 SEER registry areas before the confirmation of SPBC could be missed. Third, this study lack of detailed clinical treatments to support the association of initial cancer and SPBC. Forth, we could not adjust for some potential confounding risk factors for cancer, such as the family history of cancer, and reproductive or lifestyle factors of the patients in the present work. Also, individual genetic factors were not available; therefore, further mechanisms between primary cancers and SPBC cannot be addressed.

## Conclusions

Although cancer history has been considered as a risk factor for SPBC, further exploration reveals that the risk of developing SPBC varies by sex (female: breast, skin melanoma, thyroid, and corpus uteri; male: breast, and leukemia), age at onset of primary cancer (<50 years: breast, and thyroid; ≥50 years: breast, skin melanoma, thyroid, and corpus uteri), and race (White: breast, skin melanoma, and thyroid; Black: breast; API: breast, corpus uteri, ovary, bladder, and kidney). Our study suggests that more detailed recommendations are needed in the prevention and screening of SPBC among site-specific cancer survivors. Further studies are also needed to address the molecular/genomic and common epidemiological etiologies affecting the development of SPBC among patients with initial cancers.

## Supporting information

S1 Fig The age-standardized incidence of site-specific first primary cancers in the whole population. (PNG)

S2 Fig The age-standardized incidence of site-specific first primary cancers in White population. (PNG)

S3 Fig The age-standardized incidence of site-specific first primary cancers in Black population. (PNG)

S4 Fig The age-standardized incidence of site-specific first primary cancers in Asian/Pacific Islander population. (PNG)

S1 Table Trends in age-standardized rates of site-specific first primary cancer patients. (DOCX)

S2 Table Risk of female second primary breast cancer after previous malignancy stratified by initial primary site and latency period. (DOCX)

S3 Table Risk of female second primary breast cancer after previous malignancy stratified by initial primary site and radiation. (DOCX)

S1 Data (ZIP)

## Abbreviations

AAPC: average annual percentage change
API: Asian/Pacific Islander
BC: breast cancer
CI: confidence interval
ER: estrogen receptor
FPBC: first primary breast cancer
NHL: non-Hodgkin lymphoma
PR: progesterone receptor
SEER: the Surveillance, Epidemiology, and End Results
SIR: standardized incidence ratio
SPBC: second primary breast cancer
