# Supplementary figures and images for: Risk of second primary breast cancer among cancer survivors: Implications for prevention and screening practice
Source: PLoS One. 2020 Jun 4;15(6):e0232800. doi: 10.1371/journal.pone.0232800 (PMC7272050; doi:10.1371/journal.pone.0232800)

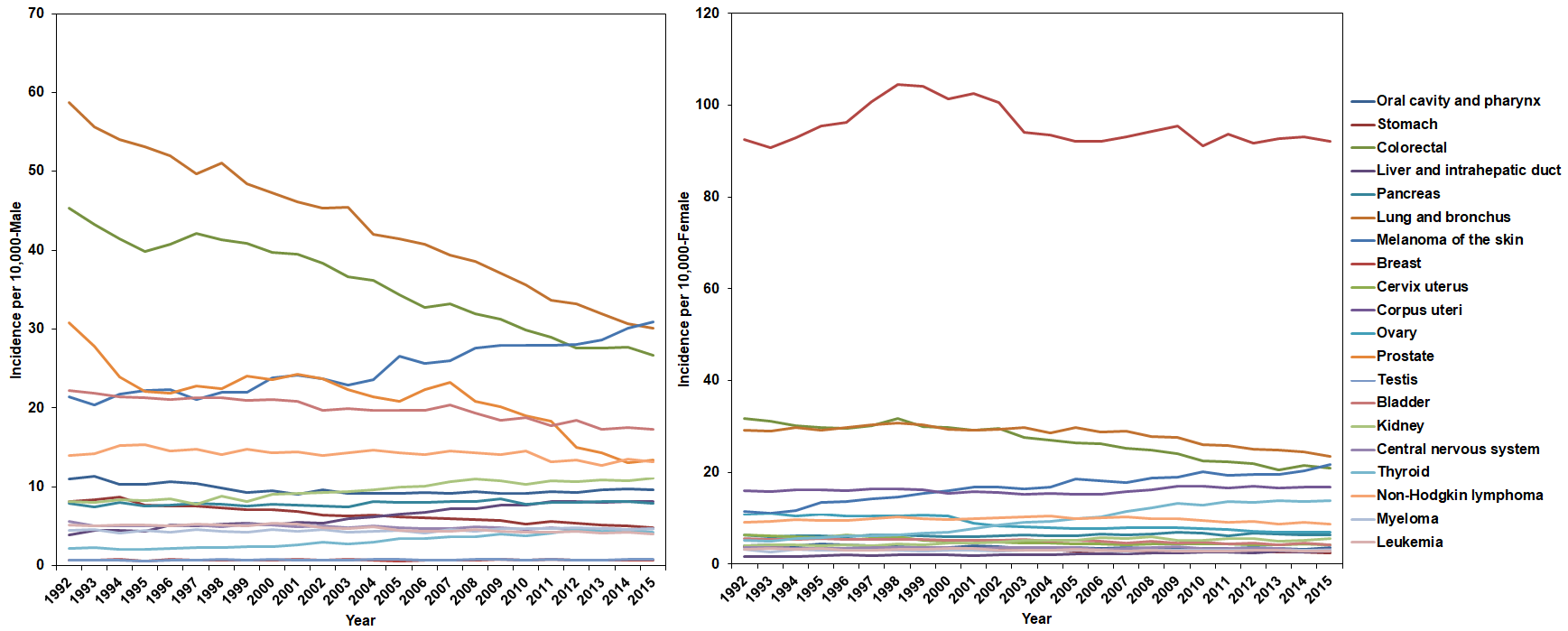

Supplement: S1 Fig — (PNG) [file pone.0232800.s001.png]

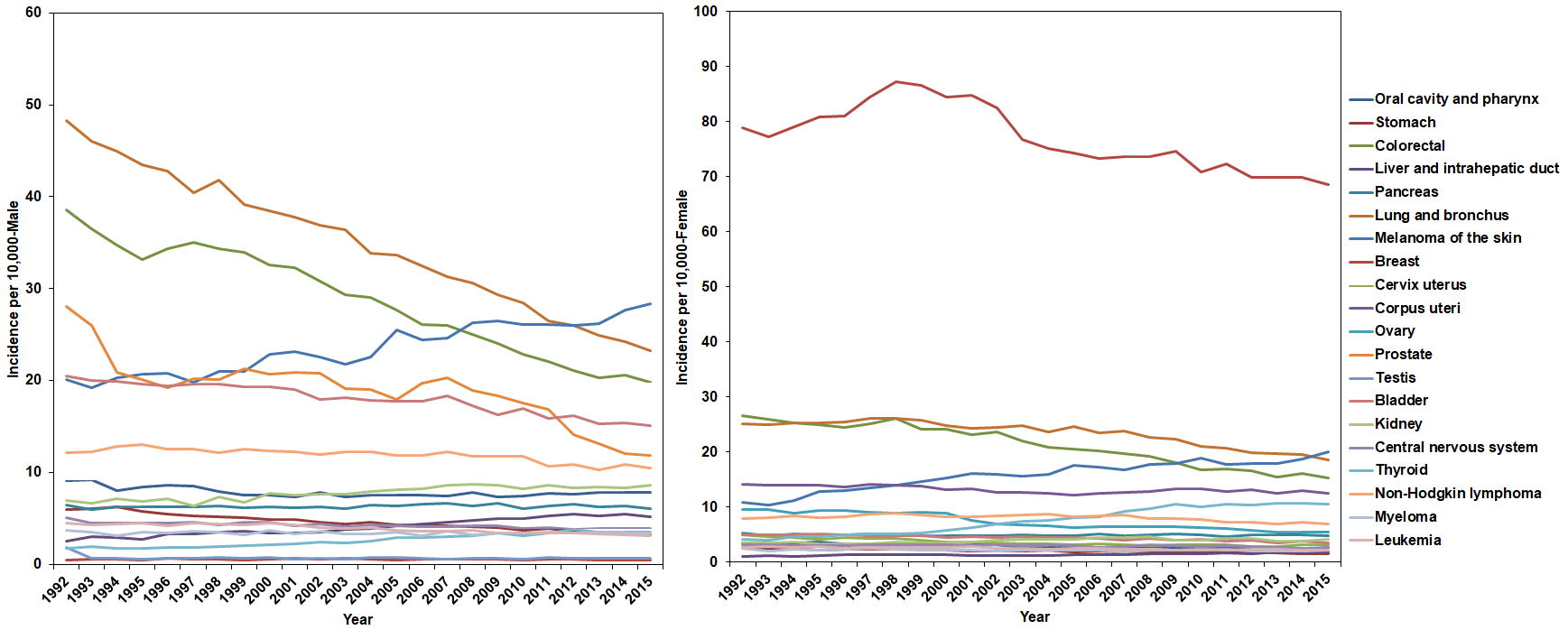

Supplement: S2 Fig — (PNG) [file pone.0232800.s002.png]

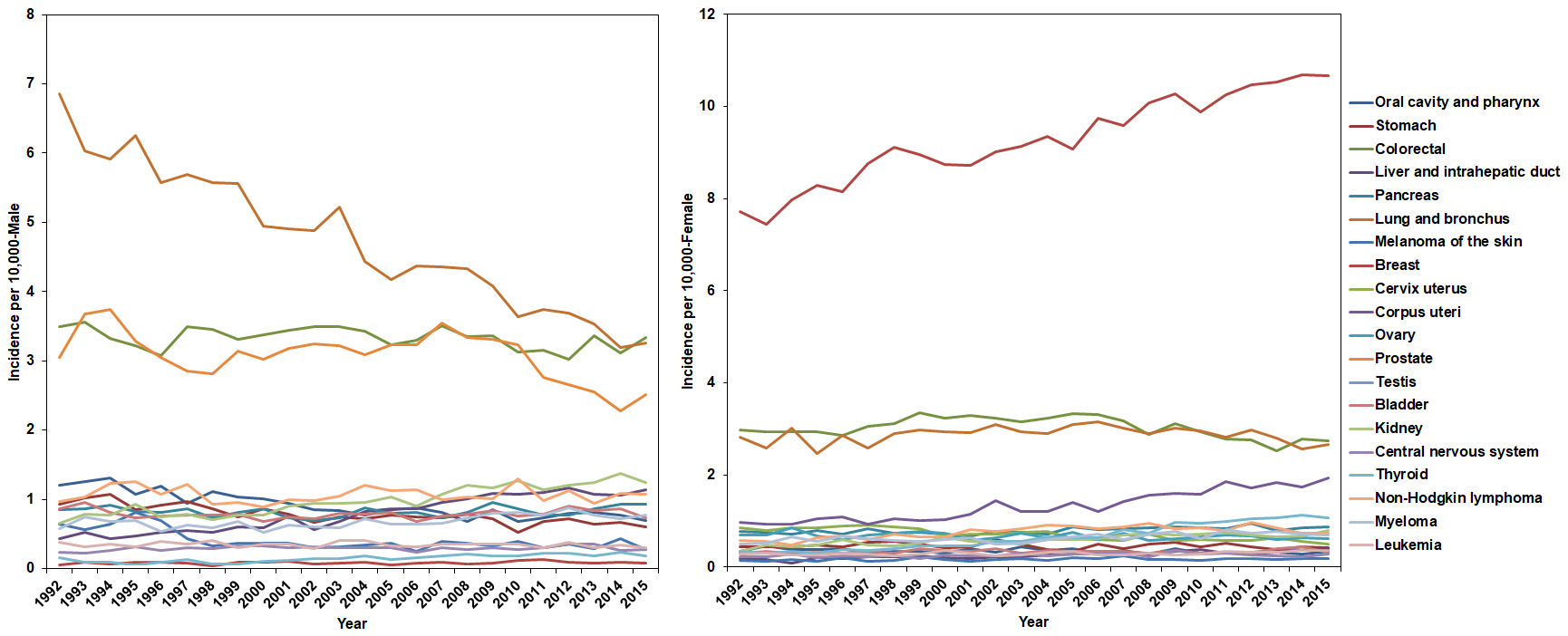

Supplement: S3 Fig — (PNG) [file pone.0232800.s003.png]

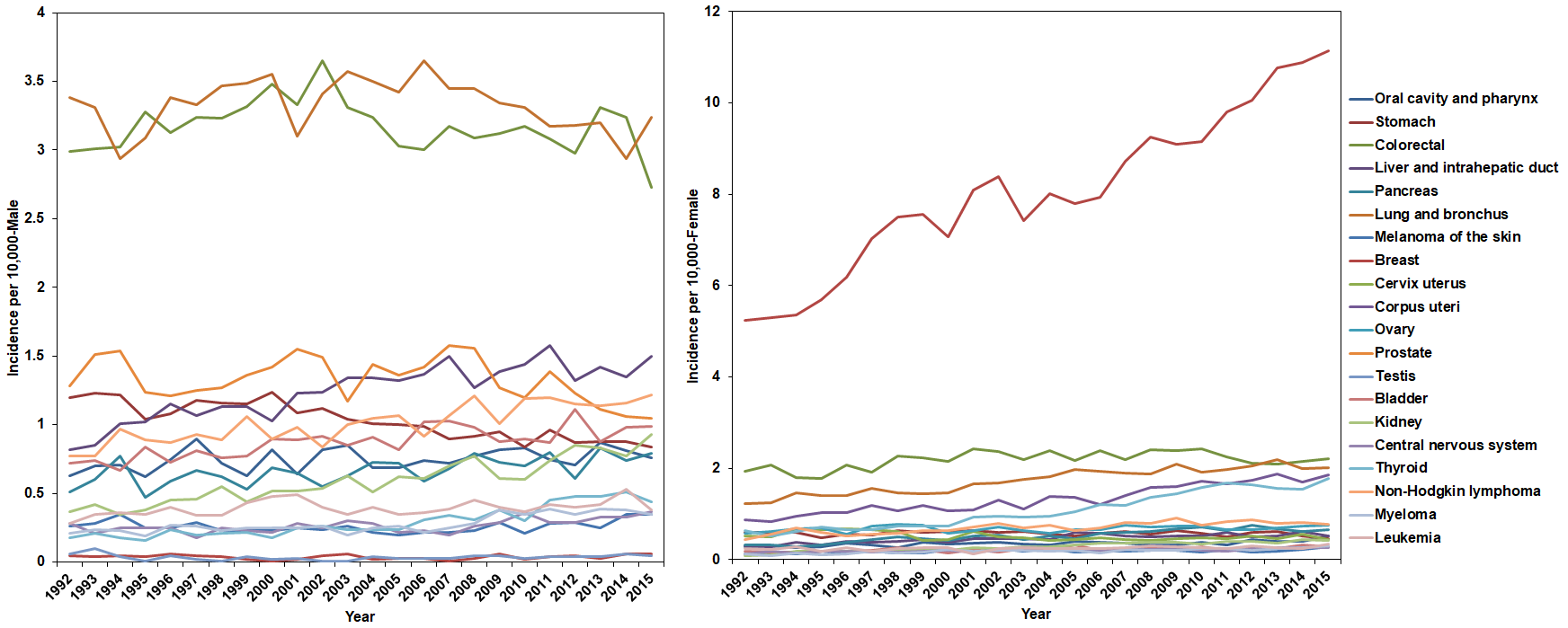

Supplement: S4 Fig — (PNG) [file pone.0232800.s004.png]
