# Supplementary material for: Risk of second primary breast cancer among cancer survivors: Implications for prevention and screening practice
Source: PLoS One. 2020 Jun 4;15(6):e0232800. doi: 10.1371/journal.pone.0232800 (PMC7272050; doi:10.1371/journal.pone.0232800)
Supplement: S1 Table — (DOCX) [file pone.0232800.s005.docx]

**Table S1. Trends in age-standardized rates of site-specific first primary cancer patients**

|  | **Male** | | | |  | **Female** | | | |
| --- | --- | --- | --- | --- | --- | --- | --- | --- | --- |
|  | **Whole Population** | **White** | **Black** | **API** |  | **Whole Population** | **White** | **Black** | **API** |
| **Oral cavity and pharynx** | -0.7 (-1.0, -0.4) | -0.7 (-1.0, -0.3) | -2.5 (-3, -1.9) | 0.6 (0, 1.2) |  | -1.0 (-1.2, -0.7) | -1.4 (-1.7, -1.1) | -1.0 (-1.8,-0.2) | 1.8 (1.0, 2.5) |
| **Stomach** | -2.3 (-2.5, -2.2) | -2.6 (-2.8, -2.5) | -2.0 (-2.5, -1.5) | -1.6 (-2.0,-1.3) |  | -1.4 (-1.7, -1.1) | -2.1 (-2.4, -1.8) | -0.3 (-1.1, 0.5) | -0.2 (-0.8, 0.3) |
| **Colorectal** | -2.3 (-3.0, -1.7) | -2.9 (-3.5, -2.3) | -0.3 (-0.5, 0) | 0.1 (-1.6, 1.9) |  | -1.8 (-2.1, -1.5) | -2.4 (-2.7, -2.0) | -0.5 (-1.0, 0) | 0.7 (0, 1.5) |
| **Liver and intrahepatic duct** | 3.0 (2.5, 3.5) | 2.9 (2.2, 3.6) | 4.4 (3.3, 5.4) | 2.0 (1.1, 2.9) |  | 2.4 (2.1, 2.7) | 2.3 (0.5, 4.2) | 3.9 (3.0, 4.7) | 2.5 (1.6, 3.5) |
| **Pancreas** | 0.3 (0.1, 0.4) | 0.1 (-0.1, 0.3) | 0.2 (-0.3, 0.8) | 1.3 (0.6, 2.0) |  | 0.3 (0.1, 0.5) | -0.1 (-0.3, 0.1) | 0.8 (0.4, 1.2) | 2.9 (1.3, 4.4) |
| **Lung and bronchus** | -2.8 (-3.0, -2.6) | -3.1 (-3.2, -2.9) | -2.9 (-3.2, -2.7) | -0.2 (-0.8, 0.3) |  | -1.0 (-1.2, -0.7) | -1.3 (-1.6, -0.9) | 0 (-0.6, 0.6) | 2.2 (1.6, 2.9) |
| **Melanoma of the skin** | 1.7 (1.5, 1.9) | 1.6 (1.4, 1.8) | -2.2 (-6.8, 2.6) | 0.6 (-0.9, 2.1) |  | 2.8 (2.3, 3.3) | 2.7 (2.1, 3.2) | 0.9 (-0.2, 1.9) | 2.0 (0.9, 3.2) |
| **Breast** | -0.1 (-0.5, 0.4) | -0.3 (-0.8, 0.3) | 1.2 (-0.4, 2.7) | 0.3 (-2.0, 2.7) |  | 0.1 (-0.3, 0.6) | -0.5 (-0.9, -0.1) | 1.4 (1.3, 1.6) | 3.7 (2.7, 4.7) |
| **Cervix uterus** | - | - | - | - |  | -1.8 (-2.3, -1.3) | -2.0 (-2.5, -1.5) | -2.3 (-2.8,-1.7) | -1.2 (-2.0, -0.4) |
| **Corpus uteri** | - | - | - | - |  | 0.2 (-0.3, 0.7) | -0.5 (-1.0, 0.1) | 3.4 (2.9, 3.9) | 3.3 (2.9, 3.8) |
| **Ovary** | - | - | - | - |  | -2.3 (-2.6, -1.9) | -2.3 (-3.3, -1.3) | -0.7 (-1.2, -0.1) | 0.5 (-0.1, 1.0) |
| **Prostate** | -3.8 (-6, -1.5) | -4.0 (-5.2, -2.7) | -1.4 (-3.8,1.1) | -1.1 (-2.2, 0.1) |  | - | - | - | - |
| **Testis** | 0.6 (0.1, 1.1) | -3.9 (-5.7, -2.1) | - | -2.1 (-7.1, 3.2) |  | - | - | - | - |
| **Bladder** | -1.1 (-1.2, -0.9) | -1.3 (-1.6, -1.1) | 0.1 (-0.4, 0.6) | 1.3 (0.8, 1.9) |  | -1.1 (-1.3, -0.9) | -1.5 (-1.7, -1.3) | 0.5 (-0.2, 1.2) | 2.1 (1.1, 3.2) |
| **Kidney** | 1.5 (1.1, 1.9) | 1.1 (0.6, 1.5) | 2.8 (2.4, 3.3) | 3.7 (3.1, 4.4) |  | 1.4 (0.8, 1.9) | 0.5 (-0.5, 1.5) | 2.3 (1.7, 3.0) | 7.3 (3.9, 10.8) |
| **Central nervous system** | -0.6 (-0.8, -0.4) | -0.9 (-1.1, -0.7) | 0.4 (-0.2, 1.1) | 1.8 (0.9, 2.6) |  | -0.3 (-0.5, 0) | -0.6 (-0.9, -0.3) | 0.8 (-0.1, 1.8) | 2.0 (1.3, 2.8) |
| **Thyroid** | 2.9 (1.7, 4.2) | 3.4 (2.6, 4.3) | 4.0 (2.7, 5.4) | 5.0 (4.2, 5.9) |  | 4.5 (3.9, 5.0) | 4.5 (4.1, 5.0) | 6.4 (5.8, 6.9) | 5.2 (4.2, 6.2) |
| **Non-Hodgkin lymphoma** | -0.5 (-0.9, -0.1) | -0.8 (-1.2, -0.5) | 0 (-0.7, 0.6) | 1.7 (1.2, 2.1) |  | -0.2 (-0.5, 0.1) | -0.7 (-1.1, -0.3) | 1.8 (0.7, 2.9) | 2.0 (1.4, 2.7) |
| **Myeloma** | 0.3 (0.1, 0.6) | -0.1 (-0.4, 0.2) | 1.2 (0.6, 1.9) | 2.0 (-1.2, 5.3) |  | 0.3 (-0.1, 0.6) | -0.4 (-0.8, -0.1) | 1.4 (0.7, 2.0) | 3.4 (2.4, 4.3) |
| **Leukemia** | -1.0 (-1.5, -0.6) | -1.4 (-1.8, -1.0) | -0.4 (-1.0, 0.3) | 0.9 (0.1, 1.7) |  | -0.7 (-0.9, -0.4) | -1.1 (-1.4, -0.9) | 0.9 (0.1, 1.6) | 1.1 (0.1, 2.0) |

Abbreviations: API: Asian/Pacific Islander.
