# Supplementary material for: Risk of second primary breast cancer among cancer survivors: Implications for prevention and screening practice
Source: PLoS One. 2020 Jun 4;15(6):e0232800. doi: 10.1371/journal.pone.0232800 (PMC7272050; doi:10.1371/journal.pone.0232800)
Supplement: S2 Table — (DOCX) [file pone.0232800.s006.docx]

**Table S2. Risk of female second primary breast cancer after previous malignancy stratified by initial primary site and latency period**

|  | **6-11 months** | | |  | **12-59 months** | | |  | **60-119 months** | | |  | **≥120 months** | | |
| --- | --- | --- | --- | --- | --- | --- | --- | --- | --- | --- | --- | --- | --- | --- | --- |
| **Initial primary site** | **SIR** | **95% CI** | |  | **SIR** | **95% CI** | |  | **SIR** | **95% CI** | |  | **SIR** | **95% CI** | |
| Oral cavity and pharynx | 0.76 | 0.49 | 1.11 |  | 0.95 | 0.82 | 1.10 |  | 0.94 | 0.78 | 1.12 |  | 0.86 | 0.69 | 1.06 |
| Stomach | 0.54 | 0.28 | 0.95 |  | 0.90 | 0.71 | 1.11 |  | 0.84 | 0.61 | 1.13 |  | 0.67 | 0.43 | 1.01 |
| Colon and rectum | 0.91 | 0.79 | 1.03 |  | 0.95 | 0.90 | 1.00 |  | 0.99 | 0.93 | 1.06 |  | 0.93 | 0.87 | 1.00 |
| Liver and intrahepatic duct | 0.91 | 0.43 | 1.67 |  | 0.75 | 0.49 | 1.10 |  | 0.66 | 0.28 | 1.29 |  | 0.41 | 0.05 | 1.48 |
| Pancreas | 0.41 | 0.20 | 0.75 |  | 0.82 | 0.59 | 1.10 |  | 1.34 | 0.85 | 2.02 |  | 1.48 | 0.79 | 2.53 |
| Lung and bronchus | 0.76 | 0.64 | 0.90 |  | 0.90 | 0.82 | 0.97 |  | 1.06 | 0.95 | 1.19 |  | 0.90 | 0.76 | 1.06 |
| Melanoma of the skin | 1.12 | 0.94 | 1.33 |  | 1.15 | 1.07 | 1.22 |  | 1.09 | 1.01 | 1.16 |  | 0.98 | 0.90 | 1.05 |
| Breast | 1.31 | 1.24 | 1.39 |  | 1.47 | 1.44 | 1.50 |  | 1.82 | 1.78 | 1.86 |  | 2.10 | 2.06 | 2.14 |
| Cervix uterus | 0.95 | 0.64 | 1.35 |  | 0.74 | 0.62 | 0.87 |  | 0.83 | 0.70 | 0.97 |  | 0.73 | 0.62 | 0.85 |
| Corpus uteri | 1.18 | 1.02 | 1.35 |  | 1.07 | 1.01 | 1.13 |  | 1.08 | 1.02 | 1.15 |  | 0.97 | 0.90 | 1.04 |
| Ovary | 0.70 | 0.52 | 0.93 |  | 0.98 | 0.88 | 1.09 |  | 1.10 | 0.97 | 1.24 |  | 1.05 | 0.93 | 1.19 |
| Bladder | 0.75 | 0.54 | 1.02 |  | 0.96 | 0.86 | 1.08 |  | 1.02 | 0.89 | 1.16 |  | 0.92 | 0.78 | 1.08 |
| Kidney | 0.99 | 0.72 | 1.32 |  | 1.02 | 0.90 | 1.14 |  | 1.08 | 0.94 | 1.24 |  | 1.10 | 0.93 | 1.30 |
| Central nervous system | 0.89 | 0.44 | 1.59 |  | 0.79 | 0.55 | 1.11 |  | 1.16 | 0.81 | 1.63 |  | 0.68 | 0.41 | 1.06 |
| Thyroid | 0.90 | 0.67 | 1.19 |  | 1.12 | 1.02 | 1.23 |  | 1.23 | 1.12 | 1.35 |  | 1.19 | 1.08 | 1.32 |
| Non-Hodgkin lymphoma | 0.62 | 0.46 | 0.81 |  | 0.83 | 0.76 | 0.92 |  | 0.92 | 0.82 | 1.02 |  | 1.20 | 1.07 | 1.34 |
| Myeloma | 0.78 | 0.49 | 1.17 |  | 0.70 | 0.56 | 0.85 |  | 0.84 | 0.61 | 1.12 |  | 0.79 | 0.45 | 1.28 |
| Leukemia | 0.71 | 0.48 | 1.02 |  | 0.87 | 0.75 | 1.00 |  | 0.88 | 0.74 | 1.05 |  | 0.97 | 0.78 | 1.19 |
| All sites | 1.05 | 1.01 | 1.09 |  | 1.20 | 1.18 | 1.22 |  | 1.42 | 1.40 | 1.45 |  | 1.56 | 1.53 | 1.58 |
| All sites except for breast | 0.88 | 0.83 | 0.93 |  | 0.98 | 0.95 | 1.00 |  | 1.04 | 1.01 | 1.06 |  | 0.98 | 0.95 | 1.01 |

Abbreviations: SIR: standardized incidence ratio.
