# Supplementary material for: Risk of second primary breast cancer among cancer survivors: Implications for prevention and screening practice
Source: PLoS One. 2020 Jun 4;15(6):e0232800. doi: 10.1371/journal.pone.0232800 (PMC7272050; doi:10.1371/journal.pone.0232800)
Supplement: S3 Table — (DOCX) [file pone.0232800.s007.docx]

**Table S3. Risk of female second primary breast cancer after previous malignancy stratified by initial primary site and radiation**

|  | **No Radiation** | | |  | **Radiation** | | |
| --- | --- | --- | --- | --- | --- | --- | --- |
| **Initial primary site** | **SIR** | **95% CI** | |  | **SIR** | **95% CI** | |
| Oral cavity and pharynx | 0.95 | 0.84 | 1.08 |  | 0.86 | 0.74 | 1.00 |
| Stomach | 0.82 | 0.69 | 0.97 |  | 0.66 | 0.39 | 1.04 |
| Colorectal | 0.96 | 0.93 | 1.00 |  | 0.86 | 0.76 | 0.97 |
| Liver and intrahepatic duct | 0.72 | 0.52 | 0.97 |  | 1.05 | 0.29 | 2.69 |
| Pancreas | 0.88 | 0.68 | 1.12 |  | 0.85 | 0.54 | 1.27 |
| Lung and bronchus | 0.95 | 0.89 | 1.02 |  | 0.81 | 0.72 | 0.92 |
| Melanoma of the skin | 1.08 | 1.04 | 1.12 |  | 1.30 | 0.60 | 2.47 |
| Breast | 1.66 | 1.63 | 1.69 |  | 1.83 | 1.80 | 1.85 |
| Cervix uterus | 0.77 | 0.68 | 0.86 |  | 0.79 | 0.69 | 0.91 |
| Corpus uteri | 1.05 | 1.01 | 1.10 |  | 1.05 | 0.97 | 1.13 |
| Ovary | 1.01 | 0.94 | 1.08 |  | 1.17 | 0.68 | 1.88 |
| Bladder | 0.96 | 0.89 | 1.03 |  | 1.00 | 0.55 | 1.68 |
| Kidney | 1.05 | 0.97 | 1.13 |  | 1.12 | 0.45 | 2.31 |
| Central nervous system | 1.04 | 0.79 | 1.35 |  | 0.73 | 0.53 | 0.99 |
| Thyroid | 1.15 | 1.07 | 1.24 |  | 1.18 | 1.09 | 1.28 |
| Non-Hodgkin lymphoma | 0.87 | 0.81 | 0.93 |  | 1.07 | 0.96 | 1.20 |
| Myeloma | 0.75 | 0.63 | 0.88 |  | 0.75 | 0.53 | 1.03 |
| Leukemia | 0.88 | 0.80 | 0.97 |  | 0.85 | 0.42 | 1.52 |
| All sites | 1.23 | 1.22 | 1.24 |  | 1.59 | 1.56 | 1.61 |
| All sites except for breast | 0.99 | 0.97 | 1.01 |  | 0.98 | 0.94 | 1.01 |

Abbreviations: SIR: standardized incidence ratio.
